# Analysis of bulk RNA-seq data from sepsis patients reveals sepsis-associated lncRNAs and targeted cell death-related genes contributing to immune microenvironment regulation

**DOI:** 10.3389/fimmu.2023.1026086

**Published:** 2023-02-02

**Authors:** Yanwei Cheng, Lijun Xu, Jiaoyang Wang, Xue Cao, Dong Chen, Peirong Zhang, Lei Yang, Lijie Qin

**Affiliations:** ^1^ Department of Emergency, Henan Provincial People’s Hospital, People’s Hospital of Zhengzhou University, People’s Hospital of Henan University, Zhengzhou, China; ^2^ Department of Rheumatology and Immunology, Henan Provincial People’s Hospital, People’s Hospital of Zhengzhou University, People’s Hospital of Henan University, Zhengzhou, China; ^3^ Wuhan Ruixing Biotechnology Co., Ltd, Wuhan, China

**Keywords:** sepsis, immune cells, cell death genes, lncRNAs, co-expression network

## Abstract

**Background:**

Sepsis is a life-threatening organ dysfunction syndrome that leads to the massive death of immune cells. Long non-coding RNAs (lncRNAs) have been reported to exert key regulatory roles in cells. However, it is unclear how lncRNAs regulate the survival of immune cells in the occurrence and development of sepsis.

**Methods:**

In this study, we used blood whole transcriptome sequencing data (RNA-seq) from normal controls (Hlty) and patients with uncomplicated infection (Inf1 P), sepsis (Seps P), and septic shock (Shock P), to investigate the fraction changes of immune cell types, expression pattern of cell death-related genes, as well as differentially expressed lncRNAs. Association network among these factors was constructed to screen out essential immune cell types, lncRNAs and their potential targets. Finally, the expression of lncRNAs and cell death genes in sepsis patients were validated by qRT-PCR.

**Results:**

In this study, we found fifteen immune cell types showed significant fraction difference between Hlty and three patient groups. The expression pattern of cell death-related genes was also dysregulated in Hlty compared with patient groups. Co-expression network analysis identified a key turquoise module that was associated with the fraction changes of immune cells. We then identified differentially expressed lncRNAs and their potential targets that were tightly associated with the immune cell dysregulation in sepsis. Seven lncRNAs, including LINC00861, LINC01278, RARA-AS1, RP11-156P1.3, RP11-264B17.3, RP11-284N8.3 and XLOC_011309, as well as their co-expressed cell death genes, were finally identified, and we validated two lncRNAs (LINC00861 and LINC01278) and four mRNA targets using qRT-PCR in sepsis samples.

**Conclusion:**

The global analysis of cell death-related genes in the occurrence and development of sepsis was carried out for the first time, and its expression regulation mode was displayed. The expression pattern of sepsis-associated lncRNAs were analyzed and identified, and the lncRNAs were significantly related to the change of immune cell proportion. We highlight the important roles of lncRNAs and their potential targets in the regulation of immune cell fraction changes during sepsis progression. The identified lncRNAs and their target genes may become new biomarkers and therapeutic targets of sepsis.

## Introduction

Sepsis remains a common and life-threatening clinical disease with high morbidity and mortality due to early-stage uncontrolled inflammation together with late-stage protracted immunosuppression (1). Annually, approximately 48.9 million people are affected by sepsis, more than 11 million of those affected die, and one sixth of sepsis survivors experience significant functional limitations (2, 3). Recent decades have seen the remarkable progress of biomarker-guided therapy targeting the excessive inflammatory response in the early stage of sepsis (4, 5). However, increasing evidence has shown that most patients dying of sepsis are actually the result of a substantially impaired activation of the immune response that is due to a cytokine storm (6, 7). Significant reductions in the number of monocytes, macrophages, natural killer (NK) cells, T cells, B cells and follicular dendritic (DC) cells were observed in the circulation of patients with sepsis (8–11). The reduction of immune cells combined with the resultant immunosuppressive effect on surviving immune cells contribute to the severe immunoparalysis (7, 12), which is strongly related to severity and mortality of sepsis. With this background in mind, biomarker-guided therapy targeting the immune cell-death pathway may significantly improve prognosis of sepsis. At present, the mechanism of how sepsis leads to extensive immune cells death constitutes an important focus of research, while is still not fully understood.

Long non-coding RNAs (lncRNAs) are a class of non-protein-coding transcripts with an arbitrary length of larger than 200 nucleotides (13). It has now been established that lncRNAs exert key regulatory roles in the innate and adaptive immune response system, thereby contributing to the development and progression of diverse diseases, including sepsis (14–16). Some lncRNAs, such as lncRNA Cox2, lncRNA EPS and lncRNA Morrbid, can regulate inflammatory gene expression programs of innate immune cells, as well as the development and homeostasis (17–19). In the adaptive immune system, lncRNAs have mainly been shown to regulate the polarization and effector function of CD4 T cells (20–22). Liu and his colleagues recently demonstrated that lncRNA NEAT1 promoted apoptosis and inflammation in LPS‐induced H9c2 cells by targeting miR‐590‐3p, providing new strategies for the treatment of sepsis (23). Likewise, Li et al. provided experimental evidence that lncRNA PCED1B-AS1 interacted with miR-155 to modulate macrophage apoptosis and autophagy in tuberculosis (24). However, little is known regarding the mechanisms of how lncRNAs regulate the survival of immune cells in sepsis, which may impede further research into treatments targeting immune microenvironment regulation in sepsis.

In the present study, we aim to (1) reveal the fraction changes of immune cell types in varying degrees of sepsis based on a public RNA-seq data; (2) profile the pattern of cell death-related genes and lncRNAs in sepsis and septic shock; (3) construct a co-disturbed network between immune microenvironment associated lncRNAs and cell death genes; (4) identify several potential lncRNAs and targets associated with the immune cell dysregulation in blood samples from sepsis and septic shock patients; finally elucidate the preliminary mechanism by which lncRNAs regulate immune cells in response to sepsis. These findings will improve our understanding on the involvement of lncRNAs in the regulation of immune cells in sepsis and contribute to the development of new targets for sepsis treatment.

## Materials and methods

### Retrieval and process of public data

GSE154918 deposited by Herwanto (25) were downloaded from Sequence Read Archive (SRA) (https://www.ncbi.nlm.nih.gov/geo/query/acc.cgi?acc=GSE154918). GSE154918 included 105 peripheral blood samples from patients with uncomplicated infections (Inf1 P), sepsis patients (Seps P), septic shock patients (Shock P), and healthy controls (Hlty). SRA Run files were converted to fastq format with NCBI SRA Tool fastq-dump. The raw reads were trimmed of low-quality bases using a FASTX-Toolkit (v.0.0.13). Then, the clean reads were evaluated using FastQC (http://www.bioinformatics.babraham.ac.uk/projects/fastqc).

### Reads alignment and differentially expressed gene analysis

The high-quality clean reads were aligned to the human GRch38 genome by TopHat2 (26) allowing 4 mismatches. Uniquely mapped reads were ultimately used to calculate read number and reads per kilobase of exon per million fragments mapped (RPKM) for each gene. The expression levels of genes were evaluated using RPKM. The differential expression of genes (DEGs) was analyzed by the software DEseq2 (27) (https://bioconductor.org/packages/release/bioc/html/DESeq2.html), which can be used to analyze the differential expression between two or more samples. DEseq2 modeled the original reads and used the scale factor to explain the difference of Library depth. Then, DEseq2 estimated the gene dispersion, and reduced these estimates to generate more accurate dispersion estimates, so as to model the reads count. Finally, the model of negative binomial distribution was fitted by DEseq2, and the hypothesis was tested by Wald test or likelihood ratio test. The analysis results were analyzed based on the fold change (FC ≥ 2 or ≤ 0.5) and false discovery rate (FDR ≤ 0.05) to determine whether a gene was differentially expressed.

### Cell-type quantification

The CIBERSORT algorithm (28) (v1.03) was used with the default parameter for estimating immune cell fractions using FPKM values of each expressed gene. A total of 22 immune cell phenotypes were analyzed in the study, including seven T cell types [CD8 T cells, naïve CD4 T cells, memory CD4 resting T cells, memory CD4 activated T cells, T follicular helper cells, and regulatory T cells (Tregs)]; naïve and memory B cells; plasma cells; resting and activated NK cells; monocytes; macrophages M0, M1, and M2; resting and activated DCs; resting and activated mast cells; eosinophils; and neutrophils.

### LncRNA prediction and direction identification

To systematically analyze the lncRNA expression pattern, we used a pipeline for lncRNAs identification similar as previously reported (29), which was constructed based on the StringTie software (30).

### WGCNA and co-expression analysis

To fully understand the expression pattern of cell death-related genes in sepsis, we applied weighted gene co-expression network analysis (WGCNA) (31) to cluster genes that have similar expression pattern with default parameters. 2045 cell death-related genes (GO: 0008219) were retrieved from the gene ontology (GO). All expressed cell death-related genes (25% with FPKM ≥ 0.5 and at least one sample with FPKM ≥ 1) in 79 samples were used as input data. Eigengenes for each clustering module were used as the representative expression pattern of genes in each module. Module–trait associations were also investigated using WGCNA.

To explore the regulatory mode between lncRNAs and their target genes, we calculated the Pearson’s correlation coefficients (PCCs) between them and classified their relation into three class: positive correlated, negative correlated and non-correlated based on the PCCs value. P-value < 0.01 and absolute PCC > 0.6 between lncRNAs and genes were picked out to draw networks by program Cytoscape (32).

### Functional enrichment analysis

To sort out functional categories of DEGs, GO terms and Kyoto Encyclopedia of Genes and Genomes (KEGG) pathways were identified using KOBAS 2.0 server (33). Hypergeometric test and Benjamini-Hochberg FDR controlling procedure were used to define the enrichment of each term.

### Other statistical analysis

Principal component analysis (PCA) analysis was performed by R package factoextra (https://cloud.r-project.org/package=factoextra) to show the clustering of samples with the first two components. The pheatmap package (https://cran.r-project.org/web/packages/pheatmap/index.html) in R was used to perform the clustering based on Euclidean distance. Student’s t-test was used for comparisons between two groups.

### Assessment of gene expression

To evaluate the validity of the potential sepsis-associated lncRNAs and the cell death-related targets in RNA-seq data, qRT-PCR was performed. Whole blood samples were obtained from 7 healthy controls, 6 sepsis patients and 6 septic shock patients, who were age-and-gender-matched. This process was approved by the ethics committee of Henan Provincial People’s Hospital, as well as the agreement of all volunteers. All the blood samples were processed immediately after collection for the isolation of peripheral blood monouclear cells (PBMCs). Total RNA was extracted from PBMCs using the TRIzol reagent (Invitrogen). cDNA synthesis was done by standard procedures, and qRT-PCR was performed on the Bio-Rad S1000 with Hieff™ qPCR SYBR^®^ Green Master Mix (Low Rox Plus; YEASEN, China) and specific primers (**Table 1**). Relative gene expression was determined by employing the 2^−ΔΔCT^ method (34) and normalized against GAPDH.

**Table 1:** Primer sequences used for qRT-PCR analysis.

LncRNA/mRNA	Forward primer	Reverse primer
LINC01278	5′-GCTATTCCAGTGCCAAGT-3′	5′-CTCCAACCATCAACATCCT-3′
LINC00861	5′-AAGGCTATGTGTAAGAAGGT-3′	5′-CTAAGAGGCTGAGGCATAC-3′
BCL11B	5′-TCATCTGCTTCCGTGTTG-3′	5′-GTGGTCTTCCTGTAGTCATAA-3′
MTOR	5′-GAATTGAAGCGTGTGAGTC-3′	5′-TCAGGTCGTGGAGAACAT-3′
IL7R	5′-GAGGAAGGCAGGAAGAGA-3′	5′-CAGGATGGAGTGAGACAAG-3′
KMT2A	5′-CATCACCATCTGCCTCATA-3′	5′-TACTTGGACTACACTACTCTG-3′

## Results

### Diversity of immune microenvironment characteristics in whole blood among healthy donors and sepsis patients

**Figure 1:**
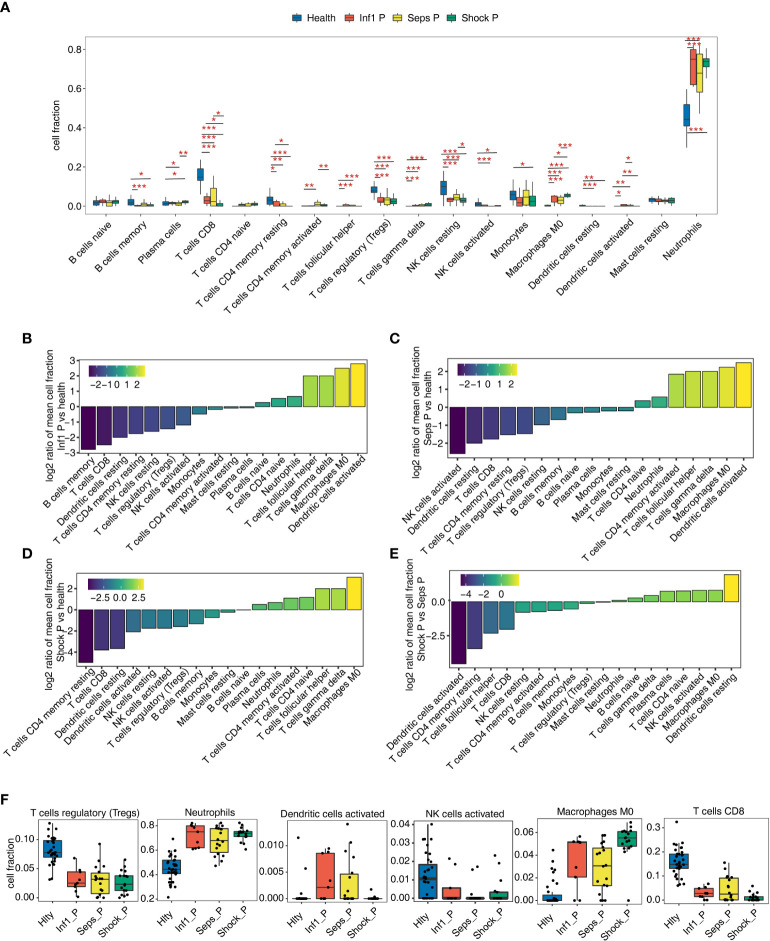
Diversity of immune microenvironment characteristics in whole blood among healthy donors and sepsis patients. **(A)** Boxplot showing fraction of each cell type in each group. The significant difference of cell fractions among healthy donors and sepsis patients was calculated using the Student’s t-test. *P ≤ 0.05, **P ≤ 0.01, ***P ≤ 0.001. **(B)** Inf1 P group relative to Hlty group rank ordered based on decreasing values of the relative frequency ratio at Inf1 P *vs.* Hlty. **(C)** Seps P group relative to Hlty group rank ordered based on decreasing values of the relative frequency ratio at Seps P *vs.* Hlty. **(D)** Shock P group relative to Hlty group rank ordered based on decreasing values of the relative frequency ratio at Shock P *vs.* Hlty. **(E)** Shock P group relative to Hlty group rank ordered based on decreasing values of the relative frequency ratio at Shock P *vs.* Seps P. **(F)** Boxplot showing cell fraction of five cell types.

A total of 79 samples were finally screened out from four groups: healthy controls (Hlty, 35 samples), uncomplicated infections (Inf1 P, 9 samples), sepsis (Seps P, 18 samples) and septic shock patients (Shock P, 17 samples). We first used CIBERSORT algorithm to characterize cell composition of the four groups, and finally identified the fractions of eighteen hematopoietic cell phenotypes. Global presentation and statistical analysis revealed that most of the immune cell types were significantly changes in at least one of the three patient groups compared with normal controls, except B cell naïve, T cell CD4 naïve, and mast cell resting (**Figure 1A**; **Figure S1A**, **Table S1**). The most abundant neutrophils cell was significantly decreased in normal controls (**Figure 1A**). Principal component analysis (PCA) of all the cell fractions also showed a clear separation between normal controls and three patient groups (**Figure S1B**), further demonstrating the changed hematopoietic cell fractions in sepsis samples. We then dedicated to explore the top changed cell fractions among these four groups. Between Inf1 P and Hlty, dendritic cells activated had highest fold change, while B cells memory had lowest fold change (**Figure 1B**). Between Seps P and Hlty, dendritic cells activated was also ranked first, and NK cells activated was ranked last (**Figure 1C**). Between Shock P and Hlty, Macrophage M0 was the top enriched cell type, while T cell CD4 memory resting was the top enriched in Htly samples (**Figure 1D**). We also analyzed the fraction changes among the three patient groups. Dendritic activated and resting cells showed highest ratio enrichment in Seps P and Shock P, respectively, suggesting the important role of dendritic cell inactivation in the progression from sepsis to septic shock (**Figure 1E**). Between Seps P and Inf1 P, activated NK cells were enriched in Inf1 P samples, and memory B cells were enriched in Seps P samples (**Figure S1C**). We then selected five cell types that showed high difference among these four groups to present the detail fraction of each sample (**Figure 1F**; **Figure S1D**). In summary, these results demonstrated that hematopoietic cell fractions were extensively regulated during the development of sepsis.

### Weighted gene co-expression analysis (WGCNA) of cell death-related genes obtained modules associated with sepsis

**Figure 2:**
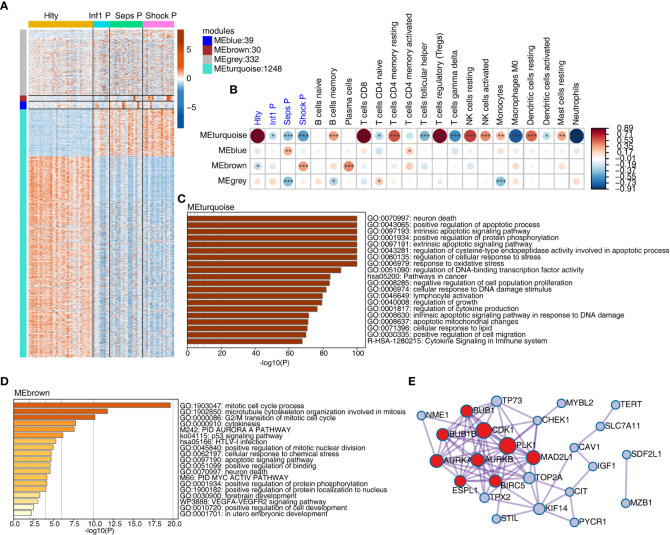
Weighted gene co-expression analysis (WGCNA) of cell death-related genes obtained modules associated with sepsis. **(A)** WGCNA analysis were performed with all expressed cell death-related genes. Heatmap showing expression profile of expressed cell death-related genes. Genes were clustered and sorted according WGCNA modules. **(B)** WGCNA module-trait associations was computed using ‘plotEigengeneNetworks’ function with all factors (disease status and cell fractions) on the x-axis used as covariates. The colors indicate Pearson’s correlation value and P-values are displayed with star. *P <=0.05; **P <=0.01; ***P <= 0.001. **(C)** Functional enrichment of genes from MEturquoise module using Metascape. **(D)** Functional enrichment of genes from MEbrown module using Metascape. **(E)** Protein-protein interaction network of genes from MEbrown.

We further investigated the underlying mechanisms of sepsis immune cell population change from the aspect of gene expression associated with cell death. We extracted 1469 expressed genes from cell-death pathway (GO: 0008219) in the gene ontology (GO) database. Differentially expressed gene (DEG) analysis of these genes revealed the dominant difference came from the comparison between Hlty and three patient groups (**Figure S2A, B**). We then used WGCNA method to classify these genes into expression modules based on their expression levels. Four expression modules were finally obtained, including grey, turquoise, blue and brown. The largest turquoise module (1248 genes) showed obvious expression difference between Hlty and other three patient samples; blue and brown modules showed higher expression patterns in Seps P and Shock P groups, respectively (**Figure 2A**). While the grey module did not show such obvious expression pattern (**Figure 2A**). We then performed module-trait analysis to construct association between identified modules and disease status or cell fractions. Turquoise module showed significant correlation (positive or negative) with four groups and most of the cell types (**Figure 2B**). Similar to the expression pattern, blue and brown modules were positively correlated with Shock P and Seps P groups, respectively (**Figure 2B**). We then analyzed the enriched functions of genes from the largest turquoise module. Besides cell death-related pathways, several other pathways were also enriched, including positive regulation of protein phosphorylation, regulation of cellular response to stress, response to oxidative stress, cytokine production and signaling, regulation of DNA-binding transcription factor activity, and pathway in cancer (**Figure 2C**). In the brown module, mitotic cell cycle-related pathways were the top three enriched pathways (**Figure 2D**), indicating that genes in this module play important role in cell cycle regulation. We then analyzed protein-protein interaction network in brown module and found the tight interaction among genes-related to cell cycle, including BUB1, BUB1B, CDK1, PLK1, AURKA, AURKB, MAD2L1, ESPL1, and BIRC5 (**Figure 2E**). Similar analysis was performed for blue module, and we found the significantly enriched cell apoptosis-related pathways (**Figure S2C**), which contained SIAH2, FBXO7, and CDC34 genes (**Figure S2D**).

### Dynamic changes of lncRNA associated with immune microenvironment regulation in healthy donors and sepsis patients with varying degrees

**Figure 3:**
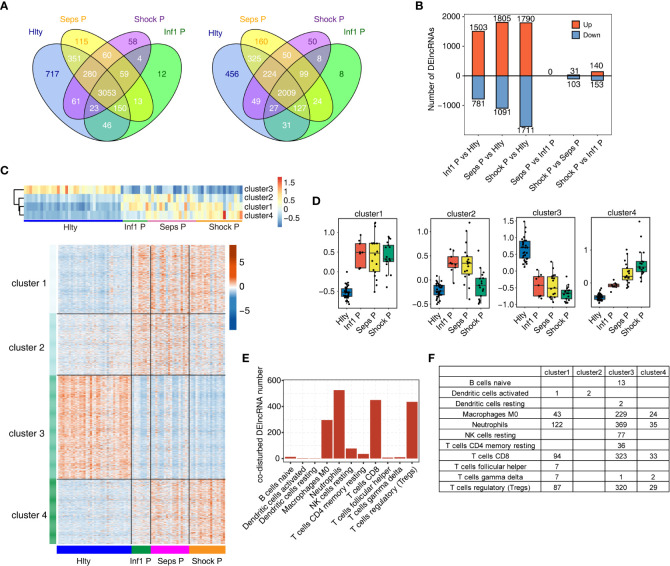
Dynamic changes of lncRNA associated with immune microenvironment regulation in healthy donors and sepsis patients with varying degrees. **(A)** Venn diagram of Known (left) and novel (right) lncRNA genes in Hlty, Inf1 P, Seps P, Shock P. At least two samples with FPKM >= 0.2 is considered to be detected in the group. **(B)** The number of differentially expressed (DE) lncRNAs among different groups. Bar plot showing the number of up-regulated and down-regulated DElncRNAs. **(C)** DElncRNAs were clustered using K-means. Heatmap in up-panel presenting 4 clusters of DE lncRNAs identified by K-means clustering and heatmap in down-panel showing expression patterns according clustered lncRNAs. **(D)** Boxplot showing cluster centers for k-mean clustering in each sample group. **(E)** The bar graph showing co-disturbed DElncRNA number of each cell type. **(F)** The statistical chart showing the distribution of co-disturbed DElncRNA number of each cell type in each cluster.

We then focused our attention on the dysregulated lncRNAs between normal controls and sepsis patients. Global lncRNA identification revealed that most lncRNAs, including known (such as *NEAT1, HULC, UCA1, MALAT1, and MIR4435-2HG*, **Table S2**) and novel lncRNAs, were overlapped among the four groups, and Hlty group also showed higher number of specific lncRNAs than other three groups (**Figure 3A**). Differentially expressed lncRNA (DElncRNA) analysis demonstrated the difference was increased by the elevated disease degree (**Figure 3B**), and the difference between Hlty and three patient groups was dominant (**Figure 3B**; **Figures S3A, B**), suggesting lncRNA expression pattern were extensively changed in sepsis patients. We then merged all the DElncRNAs and performed K-means clustering analysis. Four clusters were obtained when considering the balance between cluster number and distance. Hierarchical clustering heatmap of centroid value and the expression levels of all DElncRNAs showed cluster3 was clearly separated from other three clusters (**Figure 3C**; **Table S3**). These four clusters also showed their distinct expression pattern by plotting their centroid values using boxplot. Cluster1 was highly expressed in all three patient groups; Cluster2 was higher expressed in Inf1 P and Seps P groups and decreased in Shock P group; Cluster3 was specifically highly expressed in Hlty group; Cluster4 showed an increased pattern by the disease degree (**Figure 3D**). The dynamic expression pattern of these lncRNAs indicates they may have their specific functions in the progression of sepsis. We then attempted to construct the association between DElncRNAs and immune cell types using correlation method to further explore lncRNA functions. After filtering based on the criteria (PCC > 0.6 and P-value < 0.01), eleven immune cell types were associated with DElncRNAs; four immune cell types, including M0 macrophage, neutrophils, CD8 T cells, and Tregs, were associated with more than 200 DElncRNAs (**Figure 3E**). By classifying correlated DElncRNAs into four clusters, we found cluster3 had the largest lncRNA numbers, followed by cluster1 and cluster4 (**Figure 3F**). Meanwhile, four immune cell types, including naïve B cells, resting dendritic cells, resting NK cells, and resting CD4 memory T cells were specifically correlated with lncRNAs from cluster3 (**Figure 3F**). These results indicate that the associated DElncRNAs may contribute to the phenotype dysregulation of immune cell types.

### Construction of co-disturbed network between immune microenvironment associated lncRNAs and cell death genes

**Figure 4:**
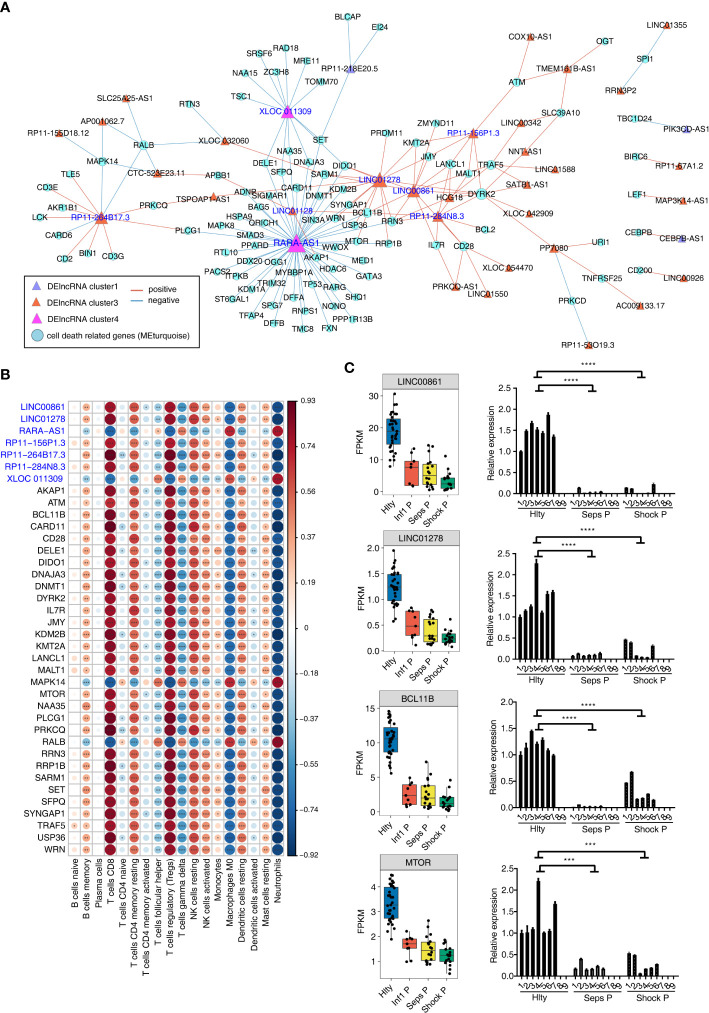
Construction of co-disturbed network between immune microenvironment associated lncRNAs and cell death genes. **(A)** The co-disturbed network between expression of immune cell related DElncRNAs and expression of cell death-related genes from three related WGCNA modules was constructed using 79 samples. |Pearson’s correlation| ≤ -0.85 or ≥ 0.95 and P-value ≤ 0.01 were retained. Circles indicate cell death-related genes and triangles indicate DE lncRNA. Lines between nodes indicate correlation between DElncRNAs and cell death-related genes. Node size indicates connections. **(B)** The dot-plot demonstrated the correlations between sample traits (each cell type) and expression of each dysregulated DElncRNAs and their target cell death related genes. Different colors indicate positive (red) or negative (blue) correlation and significant ones were labeled with star. *P ≤ 0.05; **P ≤ 0.01; ***P ≤ 0.001. **(C)** Boxplot and barplot showing expression profile of lncRNA-mRNA pairs (LINC00861-BCL11B; LINC01278-MTOR) in different groups. The left boxplots showing the expression by RNA-seq, the right showing the clinical validation by qRT-PCR. ***P ≤ 0.001, ****P ≤ 0.0001.

The canonical roles of lncRNAs are to regulate expression and functions of their targets in *cis* or *trans* manners. We have constructed the association and DElncRNAs and immune cell types, so we further investigated DElncRNAs and their potential target genes using co-expression network method. Using the cell death-related genes and DElncRNAs as input, we constructed cell death gene and DElncRNA network. Most of the involved DElncRNAs came from Cluster3, and cell death-related genes came from turquoise module (**Figure 4A**). We found two DElncRNAs from Cluster4, including XLOC_011309 and RARA_AS1, were negatively correlated with large number of genes from turquoise modules, suggesting their potential regulatory functions on their co-expressed genes (**Figure 4A**). From the co-expression network, we extracted seven DElncRNAs with high correlated gene number and their co-expressed genes to investigate their association with the eighteen immune cell types. We found the highly dysregulated immune cell types in **Figure 1A** also showed significant association with DElncRNAs and cell death-related genes. The highly associated immune cell types included CD8 T cells, Tregs, M0 Macrophages, Neutrophils and other cells (**Figure 4B**). These results indicate that the associated DElncRNAs and cell death-related genes may play important roles in the dysregulation of immune cell types in sepsis. We then presented the detail expression pattern of these seven DElncRNAs and their co-expressed potential targets. LINC00861 and LINC01278 were higher expressed in Hlty, and their co-expressed targets BCL11B and MTOR were higher expressed in Hlty (**Figure 4C**). The expression levels of other five DElncRNAs and their potential targets were shown in **Figure S4**. We propose that these seven DElncRNAs and their targets may be the key regulatory molecules of immune cell survival in the occurrence and development of sepsis, and can be treated as potential therapeutic targets.

### Validation of the expression of lncRNAs and cell death genes in healthy control and sepsis patients by qRT-PCR

We finally validated the reliable expression changes of the immune microenvironment associated lncRNAs and the co-expressed cell death-related genes in sepsis detected in GSE154918 RNA-seq data. Six sepsis patients, six septic shock patients and seven healthy controls with matched age and sex features were enrolled to extract PBMCs and perform validation experiments. Two DElncRNAs of LINC00861 and LINC01278 were selected as candidates. The expression levels of both lncRNAs in Seps P and Shock P groups were significantly lower than those of Hlty group, which were consistent to those acquired from RNA-seq in GSE154918, although there was individual variation within the same group (**Figure 4C**). Four cell death-related genes, including BCL11B, MTOR, IL7R and KMT2A, were also detected in Seps P *vs.* Hlty and Shock P *vs.* Hlty groups (**Figure 4C**; **Figures S4C, D**). All of them showed high consistency with their up-regulated changes from the RNA-seq data. Taken together, these results demonstrated the important roles of lncRNAs and their potential targets in immune microenvironment regulation during the occurrence and development of sepsis, thereby could serve as new biomarkers and targets for facilitating treatment in sepsis.

## Discussion

Sepsis is charactered by the body’s systemic immune response to infection, along with the dysfunctional and fraction-changed immune cell types (35–38). There is no doubt that extensive death of immune cells is a major driver of sepsis, and the extent of immune cell death is strongly associated with severity and mortality of sepsis. However, the mechanisms of how immune cells die so extensively in sepsis is far from clear. With the development of second-generation sequencing technology, there is increasing evidence that lncRNAs play vital roles in regulating the cell-death pathway of diverse immune cells. Based on bioinformatics technology and the public RNA-seq data of GSE154918, we here explored the mechanism through which lncRNAs regulate the cell death of immune cells in sepsis, providing new opportunities for biomarker-guided therapy targeting the immune cell-death pathway in sepsis cases.

It is well known that sepsis initiates a complex immunopathogenesis process involving both innate and adaptive immune cells (39, 40). In the present study, we systematically analyzed the composition and alternation of hematopoietic cell types between normal and sepsis specimens using whole blood transcriptome sequencing (RNA-seq) dataset GSE154918 and bioinformatics tools, and also found that fifteen immune cell types were extensively regulated during the development of sepsis, including lymphocytes, macrophages, monocytes, NK cells, neutrophils, and DCs. Numerous studies indicated that NK cells are affected during sepsis and the absolute number of circulating NK cells is markedly decreased in sepsis patients, which frequently persists for weeks (38, 41, 42). Monserrat et al. reported that CD8^+^ T cells are significantly changed by sepsis and counts of which drop significantly in sepsis patients compared with healthy subject (43). Neutrophils are essential for pathogens eradication and for sepsis survival. It was reported that patients with sepsis typically have markedly increased numbers of circulating neutrophils of various degrees of maturation (44). Our bioinformatics analysis results showed consistently changing pattern of these cells described above when comparing their fractions in Hlty group with that in sepsis groups (**Figure 1A**). In addition, we also found M0 type of macrophages were significantly upregulated in sepsis and sepsis shock patients. Previous study has demonstrated that the important antigen-presenting dendritic cells (DCs) are obviously changed in the development of sepsis at multiple levels, including the number, differentiation, and functions (45). We found the resting DCs and activate DCs showed reverse changes in septic groups (**Figures 1B, E**), suggesting their extensive dysregulation and they could be treated as novel therapeutic target during sepsis development. Meanwhile, we also observed that Tregs and γδ T cells were decreased and increased in septic groups, respectively, which were not well correlated with published studies (46, 47). We conjecture that this difference may come from the different analyzing method in our study. In summary, the dysregulated immune system greatly contributes to pathogenesis of sepsis. Further studies are needed to resolve the underlying mechanisms of immune response to sepsis at higher resolution or even at single cell level (48).

Another important discovery in this study is that we systematically investigated the expression pattern and potential functions of lncRNAs in sepsis, and deciphered their potential regulatory roles in cell-death pathway and immune cell types. Increasing evidences demonstrate that lncRNAs are essential regulators of inflammatory response and potential biomarkers of sepsis, including NEAT1, HOTAIR, UCA1 and HULC (49–51). We highlighted cell death process was associated with lncRNAs in septic transcriptome data, and identified several lncRNAs without known functions. Immune cell death plays critical roles in sepsis by releasing a large quantity of damage-associated molecular patterns (DAMPs) and inducing the dysfunction syndrome of multiple organs (10). Immune cell apoptosis is considered to be a contributing factor for immunosuppression in sepsis (52). We propose that dysregulated lncRNAs in sepsis are involved in the cell death process by modulating expression of associated genes. Among lncRNAs co-expressed with cell death genes from turquoise module, XLOC_011309 is a novel lncRNA identified for the first time; while RARA_AS1 is the antisense RNA of RARA and identified as potentially risk lncRNA that might lead to septic shock (53). Another study selected RARA_AS1 as the hub genes in mRNA-lncRNA-Pathway co-expression network in developing pediatric sepsis (54). In this study we also observed RARA_AS1 was co-expressed with large number of cell death genes and its expression was significantly higher in septic groups (**Figure 4A**; **Figure S4A**), indicating the potentially regulatory roles of RARA_AS1 in cell death in sepsis. It will be very important and has clinical values to further explore the functions and molecular mechanisms of RARA_AS1 in cell death during the development of sepsis.

Meanwhile, we constructed the relationship between lncRNAs and dysregulated immune cell fractions in sepsis for the first time. One recent study has summarized that lncRNAs emerge as key epigenetic and transcriptional regulators of immune cells and modulate the polarization and homeostasis of immune cell population (55). In sepsis, lncRNAs are recognized as potential targets for sepsis-induced cellular disorders and sepsis-induced organ failure, suggesting lncRNAs have functions in the cellular homeostasis of septic patients (56). We identified seven DElncRNAs that were correlated with the fractions of immune cell types (**Figure 4B**). We have discussed lncRNA XLOC_011309 and RARA_AS1 in the above paragraph, meanwhile the other five lncRNAs were all significantly downregulated in septic groups (**Figure 4C**; **Figure S4A**). The regulatory outcomes of lncRNAs on immune system have been extensively studied in cancer immunity, including immune evasion (57), immune cell infiltration, and immunotherapy response (58). In sepsis, very limited lncRNAs were reported to be associated with immune system dysregulation and the underlying mechanisms are largely unknown. LncRNA MALAT1 could accelerate inflammatory response by promoting neutrophil migration, and finally aggravating the progression of sepsis (59). LncRNA HOTAIRM1 is highly expressed in late phase of sepsis in a mouse model; it could induce T cell exhaustion by increasing the percentage of PD-1^+^ T cells and regulatory T cells (60). In this study, five downregulated lncRNAs in sepsis groups, including LINC00861, LINC01278, RP11−156P1.3, RP11−264B17.3, and RP11−284N8.3, were positively correlated with several immune cells, including CD8^+^ T cells, CD4^+^ memory T cells, Tregs, NK cells, and resting dendritic cells (**Figure 4B**). We infer that these lncRNAs could modulate the proportion of dysregulated immune cell types in sepsis perhaps by regulating gene expression profile and protein functions. Integrating with the association between lncRNAs and cell death genes identified in this study, it is possible that lncRNAs are potentially master regulators and the link between cell death process and immune cell population changes in sepsis.

## Conclusions

In summary, our study depicted the co-distributed network among immune cell types, cell death genes, and lncRNAs in the development of sepsis, and demonstrated the hub regulatory lncRNAs in the network. These results indicate that the identified lncRNAs may orchestrate the process of cell death and finally modulate the immune microenvironment of sepsis, although they are largely with unknown functions in sepsis. Following studies are necessary to systematically address the molecular functions and mechanisms of these dysregulated lncRNAs in sepsis, which is not deeply investigated in this study. More clinical samples should be used to further validate the dysregulated lncRNAs and their potential as septic biomarkers. In summary, the discovery contributes to the understanding of lncRNA functions in the septic immunity and the discovered lncRNAs could be served as potential therapeutic targets of sepsis in the future.

## Data availability statement

The original contributions presented in the study are included in the article/**Supplementary Material**. Further inquiries can be directed to the corresponding authors.

## Ethics statement

The studies involving human participants were reviewed and approved by the ethics committee of Henan Provincial People’s Hospital. The patients/participants provided their written informed consent to participate in this study.

## Author contributions

Conceived and designed the experiments: YC, LY and LQ. Performed the experiments and analyzed the data and investigated expression level of genes: JW, DC, PZ and XC. Wrote the manuscript: YC and LX. All authors approved the final version. All authors contributed to the article and approved the submitted version.
