# Supplementary figures and images for: Analysis of bulk RNA-seq data from sepsis patients reveals sepsis-associated lncRNAs and targeted cell death-related genes contributing to immune microenvironment regulation
Source: Front Immunol. 2023 Feb 2;14:1026086. doi: 10.3389/fimmu.2023.1026086 (PMC9932711; doi:10.3389/fimmu.2023.1026086)

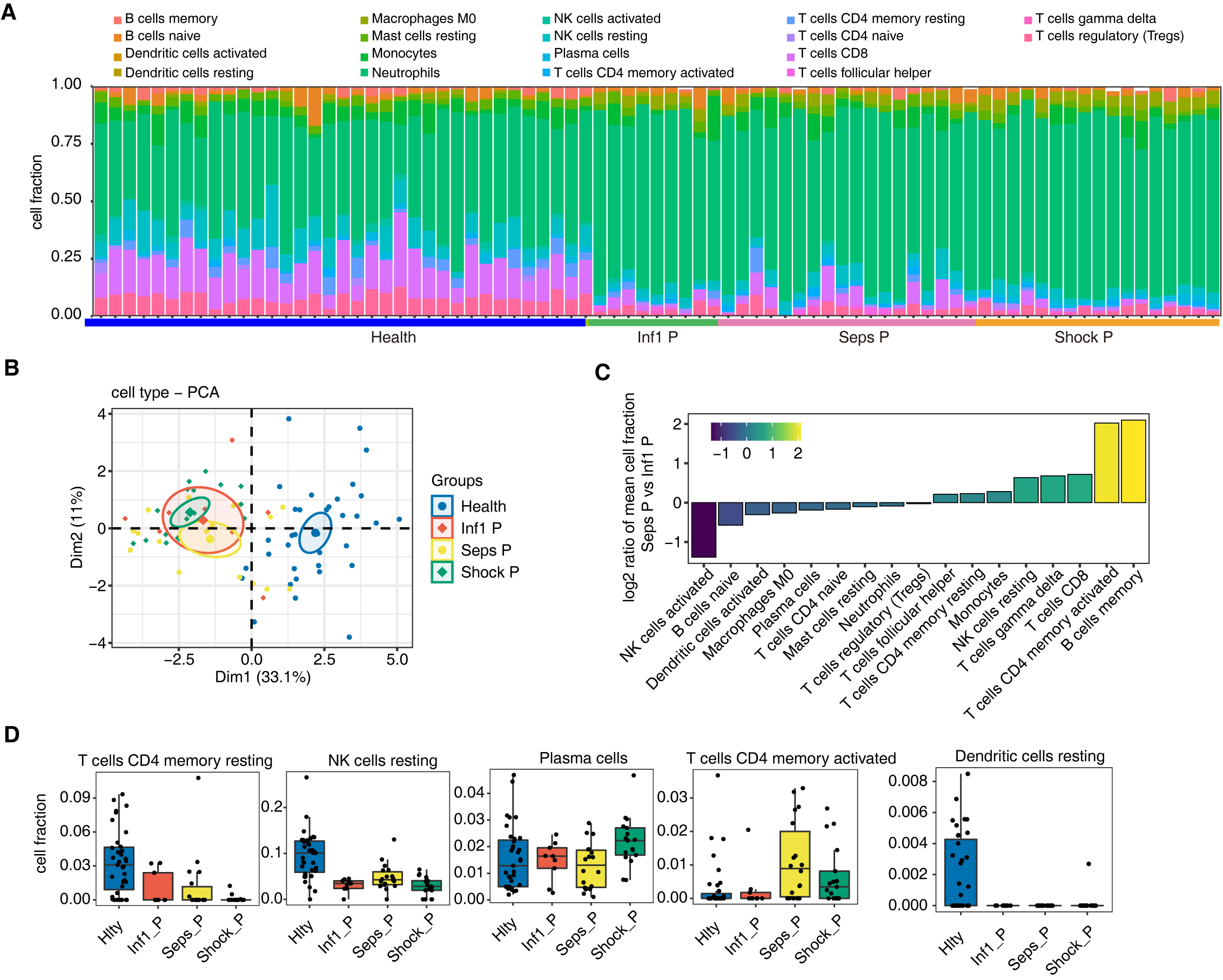

Supplement: Supplementary file 1 [file Image_1.tif]

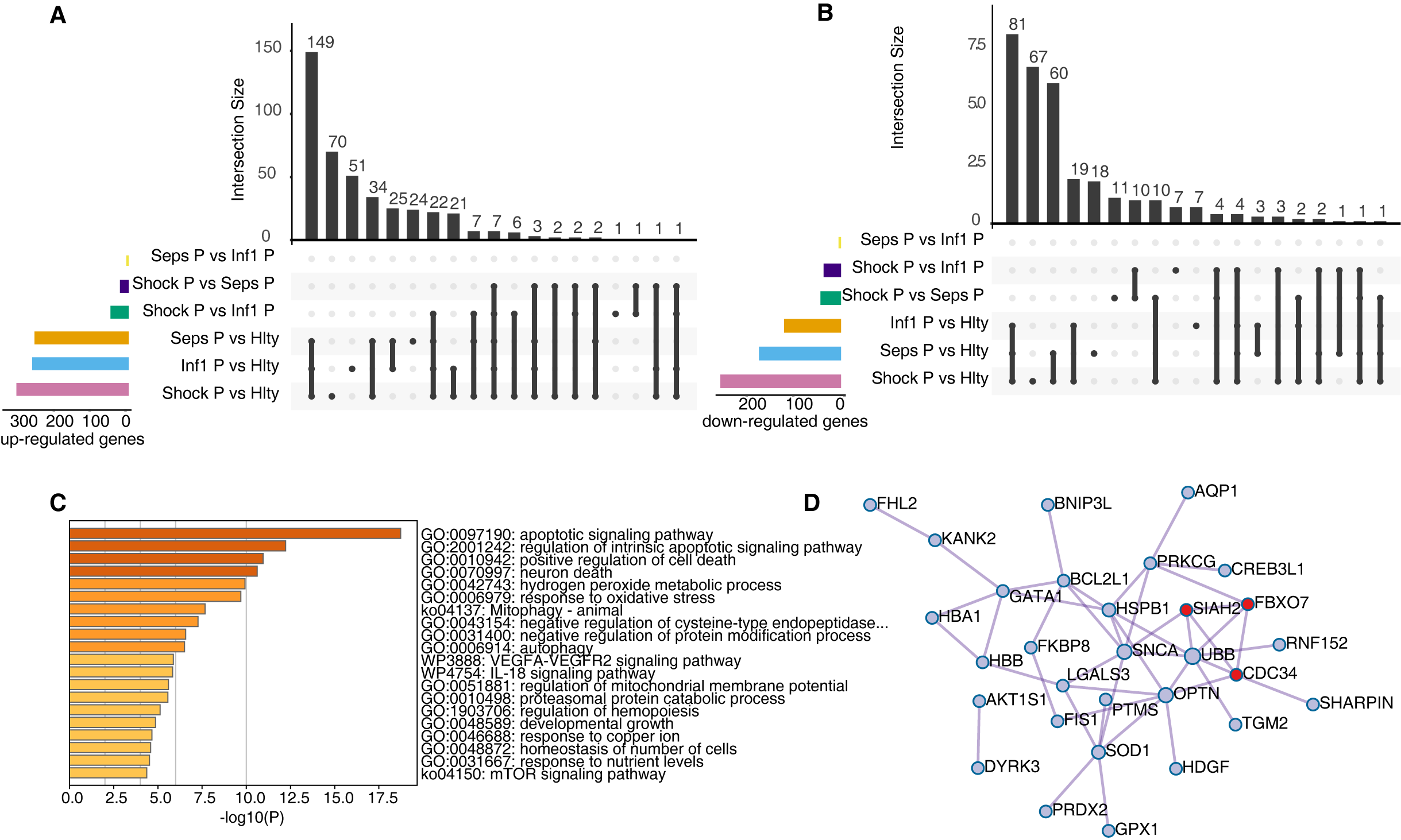

Supplement: Supplementary file 2 [file Image_2.tif]

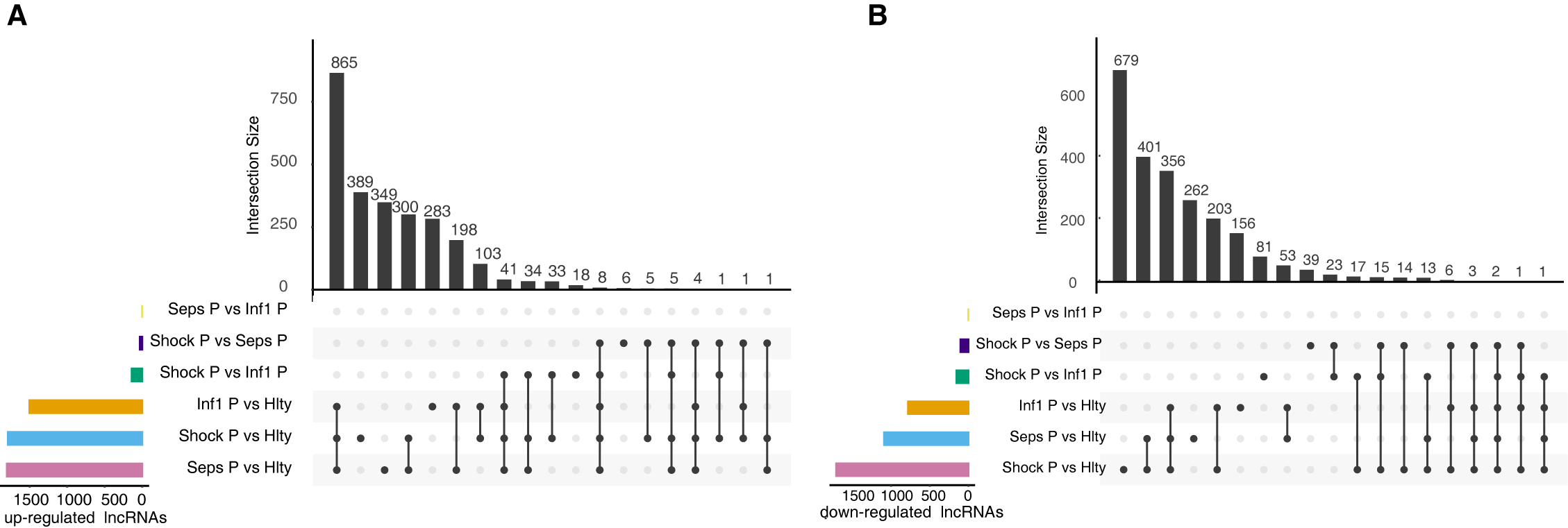

Supplement: Supplementary file 3 [file Image_3.tif]

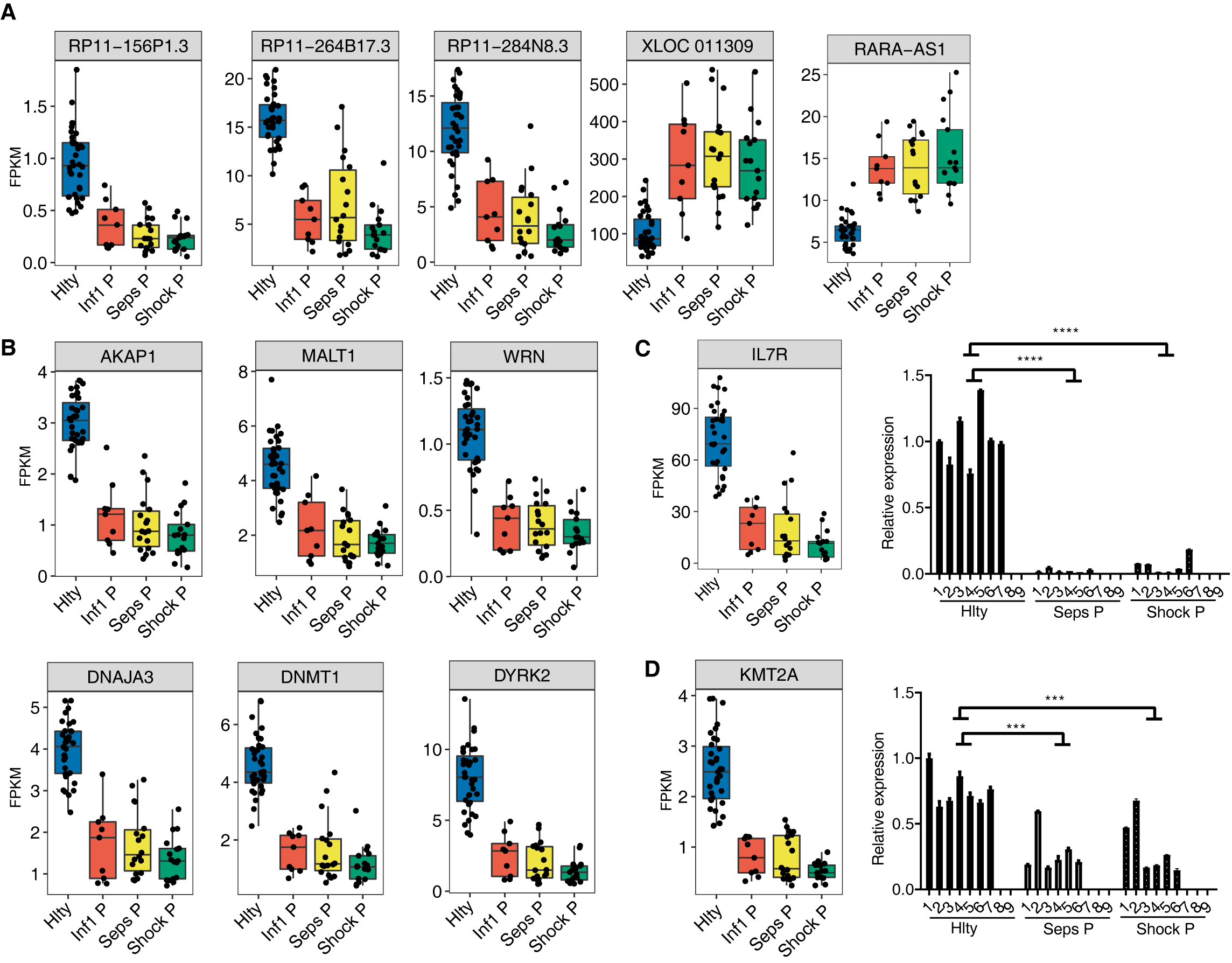

Supplement: Supplementary file 4 [file Image_4.tif]
